# The Nexus between Environmental Corporate Social Responsibility, Green Intellectual Capital and Green Innovation towards Business Sustainability: An Empirical Analysis of Chinese Automobile Manufacturing Firms

**DOI:** 10.3390/ijerph20031851

**Published:** 2023-01-19

**Authors:** Wenjie Li, Muhammad Yaseen Bhutto, Idrees Waris, Tianyang Hu

**Affiliations:** 1 School of Business Administration, Shandong University of Finance and Economics, Jinan 250014, China; 2 Business School, Shandong Jianzhu University, Jinan 250014, China; 3 Department of Management Sciences, University of Turbat, Turbat 92600, Pakistan; 4 Business School, University of Birmingham, Birmingham B15 2TT, UK

**Keywords:** environmental corporate social responsibility, green intellectual capital, green innovation, environmental sustainability, business sustainability

## Abstract

Manufacturing organizations have a pivotal role in reducing the adverse impact of global warming by adopting sustainable practices and producing environmentally-friendly products. Organizations are engaged in environmental corporate social responsibility (ECSR) and emphasize green intellectual capital (GIC), green innovative products and support for business sustainability (BUS). The current study aims to analyze the impact of organizational ECSR and GIC on green innovation (GIN) and BUS. The data for 237 participants from the manufacturing firms were analyzed via partial least square structural equation modelling (PLS-SEM). The study results revealed that ECSR and GIC are crucial for GIN and BUS. The study’s findings revealed that ECSR positively and significantly impacts green relational capital (GRC) and green structural capital (GSC). However, ECSR’s positive impact on green human capital (GHC) was insignificant. Further, the results of the mediation analysis show that GIN serves as a full mediator between GIC’s two components, GRC and GSC and a partial mediator between GHC and BUS. This study extends the environmental management literature and suggests measures for practitioners to enhance organizational capabilities in order to address environmental issues through innovative green initiatives.

## 1. Introduction

The debate on sustainable development is shifting toward the firm’s environmental management [1,2]. Managers today are fully aware of the development of environmental issues as well as how they relate to business processes [3]. As a part of business strategy, firms are taking protective and preservation measures to ensure the environment’s safety [4]. The change in business strategies has been influenced by a variety of factors, including customers’ environmental awareness [5], environmental regulation [6], environmental commitment to society [7] and corporate environmental practices [8]. These developments have encouraged businesses to introduce innovative products with a minimal environmental impact and to enhance business performance [9,10].

ECSR advocates for an organization’s efforts to consider the environmental effects of its activities and to mitigate such impacts [11]. It can have a positive environmental impact by reducing greenhouse gas emissions, saving resources, eliminating waste and preserving ecosystems [12]. However, it is important to note that the impact of ECSR can vary widely depending on the specific initiatives and practices a company adopts [13,14]. Previous studies have focused on how ECSR practices facilitates the retrieval of valuable resources [15] and improves stakeholder relationships and reactions [16]. When a company integrates sustainability into its business operations, it demonstrates a commitment to being a responsible corporate citizen [17]. The nexus between ECSR and GIC in business operations can increase green innovation by aligning the company’s values and actions with environmental and social concerns, attracting and retaining customers and fostering business sustainability [18,19].

According to Mehmood and Hanaysha [19], companies with strong commitment to ECSR tend to have higher levels of green intellectual capital. They concluded that ECSR activities can act as a catalyst for the development of green intellectual capital. Chen and Chang [20] emphasized that companies that engage in ECSR are more likely to invest in training and development programs, which can help to build green intellectual capital. Another study suggests that companies that prioritize environmental sustainability are more likely to invest in research and development related to eco-friendly products and practices, leading to an increase in GIC [21]. In addition, companies that emphasize environmental sustainability tend to have higher levels of GIC, which can lead to improved business sustainability [22]. Ali et al. [23] examined the relationship between green intellectual capital and green innovation in the manufacturing SMEs of Pakistan. The study result shows that GICs significantly increase green innovation adoption in manufacturing industry. Ullah et al. [24] highlighted the importance of the antecedents of green intellectual capital that could affect business sustainability. They indicated that green intellectual capital positively influences business sustainability. However, they have failed to explore the relationship between ECSR, GIC and GIN regarding BUS, and mainly in the context of automobile manufacturing companies. Therefore, the present study uses a comprehensive empirical framework to discuss how the relationship between ECSR and green intellectual capital affects green product innovation and business sustainability in automobile manufacturing companies in China.

One of the energy-intensive sectors that has the potential to significantly increase environmental carbon dioxide emissions is industrial manufacturing [25]. In order to reduce emissions, this industry must focus on developing policies that help the environment. Researchers have claimed that technological advancement could encourage green innovation and improve business performance while preventing environmental pollution [26,27]. In this regard, manufacturing companies can concentrate on ECSR and GIC to achieve environmental sustainability. The current study aims to test the connection between ECSR, GIC, GIN and BUS, previously ignored by researchers [28].

This study addresses three research questions. First, how does ECSR affect green intellectual capital (GIC)? Second, does GIC lead to green innovation and business sustainability? Third, does green innovation mediate the relationship between GIC and business sustainability? The current study will contribute to the existing environmental management literature by evaluating the connections between ECSR, GIC, GIN and BUS. Past studies were based on resource-based view (RBV) theory [29], institutional theory [22] and social exchange theory [24]. This study is based on the premise of the intellectual Capital-based view theory that emphasizes specific organizational elements closely related to competitive advantage and business performance. Understanding how ECSR affects an organization’s intellectual capital is crucial for promoting green innovation and business sustainability [22,30].

## 2. Literature Review

### 2.1. Environmental Corporate Responsibility and Green Intellectual Capital

Environmental management and CSR concepts served as the foundation for ECSR. A crucial and unique component of CSR is CER [31]. According to Mazurkiewicz’s research [32], corporate environmental responsibility (CER) also takes into account the environmental impact of a company’s operations, products and facilities. Companies advocating ECSR reduce resource utilization and carbon dioxide emissions, implementing eco-friendly processes that have minimal negative impact on future generations [33,34]. ECSRs are also defined as environmentally responsible business practices that go above and beyond regulatory obligations and accept responsibility for any adverse external effects of their operations [35]. Green intellectual capital, on the other hand, refers to the knowledge, expertise and innovation within a company that is related to environmental sustainability [36]. This can include factors like research and development of new green technologies, as well as the skills and knowledge of employees with regards to environmental sustainability [37]. Past studies have established positive links between ECSR and GIC [35,36,37]. Researchers have argued that companies that are committed to environmental responsibility are likely to invest in green intellectual capital, as this can help them to identify and implement more sustainable practices [38,39]. At the same time, green intellectual capital can help a company to be more effective in its efforts to reduce its environmental impact, as it can provide the knowledge and expertise needed to identify and implement the most effective sustainability measures. In a nutshell, ECSR and the support of the GIC can help businesses to introduce green innovation and achieve business sustainability. Table 1 shows the key findings of related studies.

### 2.2. Intellectual Capital-Based View Theory

**Figure 1:**
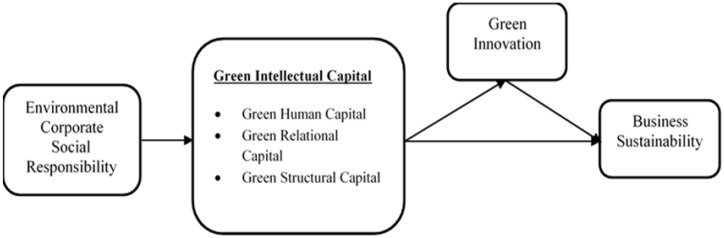
Conceptual Model.

The current research is based on the intellectual capital-based view (ICV) theory. ICV theory has been widely used by researchers as the theoretical underpinning to evaluate organizational performance [31,32,33]. This theory focuses on the specific intangible aspect of the business’s function that is vital for organizational performance [34]. Based on ICV, ECSR enhances businesses in achieving their environmental goals. Past studies have empirically established that an organization’s ability to maintain a competitive advantage depends on the effectiveness of its intellectual resources [35,36]. Despite the fact that ECSR and GIC are important for driving green innovation, little research has been conducted to demonstrate empirically how they affect green innovation and business sustainability. Therefore, the current study extends the discussion of the relationship between ECSR and GIC leading to green innovation and business sustainability. The conceptual framework is shown in Figure 1.

Past studies have indicated that businesses devoted to environmental responsibility are likely to invest in green intellectual capital since this may assist them in identifying and implementing more sustainable practices [22,43]. In addition, ECSR refers to a company’s responsibilities to its internal shareholders, top management, employees, customers, suppliers, business partners, communities and interest groups [11,15]. Companies that place a high value on external stakeholders, such as environmentally conscious customers, will usually invest more money in environmentally friendly initiatives, including GIC and green innovation [19,35]. Additionally, as corporate sustainability initiatives increase, organizations will invest more in green human capital and green relational capital [28]. To ensure business sustainability, ECSR includes the incorporation of plans and policies, such as employee development program and innovative product design. Hence, it is proposed that:

**H_1a_.**  *ECSR has a positive influence on green human capital.*

**H_1b_.**  *ECSR has a positive influence on green relational capital.*

**H_1c_.**  *ECSR has a positive influence on green structural capital.*

### 2.3. Green Intellectual Capital (GIC)

Green intellectual capital is crucial for business sustainability as it encompasses the knowledge, skills and expertise that organizations possess related to environmental and social issues [47]. However, since its introduction in 2008, green IC has not received much academic attention [25]. Businesses can produce superior performance and gain a competitive advantage as a result of rare intangible assets [47,48]. Researchers believe that green IC refers to company’s overall expertise that helps the environment and gives the company a competitive edge [49]. GIC also assesses customers’ environmental awareness and ensures strict compliance with international environmental regulations [47]. It is the intangible assets that have an impact on the sustainability of the company [48]. Researchers have assessed the effects of GIC on the sustainability of the business. Notably, Omar et al. [44] examined the link between GIC and business sustainability and the findings of the study suggested a significant relationship between GIC and business sustainability. Similarly, Ullah et al. [24] confirmed the positive relationship between GIC and business sustainability of Chinese manufacturing firms. These results indicate the importance of GIC in business sustainability. In this study, GIC has been divided into three categories: green relational capital (GRC), green structural capital (GSC) and green human capital (GHC).

#### 2.3.1. Green Human Capital (GHC)

Human capital is crucial for a company’s success and competitive advantage [47,49,50]. According to Chen and Chang [20], GHC is the individual and collective knowledge, attitude, skills, experience, creativity and commitment to green innovation possessed by employees. The impact of green human capital on business sustainability can be significant, as it can help a business to reduce its environmental impact, lower its costs and improve its reputation [23]. In addition, employees with knowledge of sustainable practices can help a business to implement more efficient processes that use fewer resources, reduce waste and minimize pollution [44]. Therefore, employees are likely to be motivated to apply their environmental knowledge to green innovation [51]. Researchers have argued that the skills and the knowledge of environmental issues possessed by employees determine the continuity of environmental practices in businesses [51,52,53]. The novelty introduced by the demand for GHC investment could significantly promote green innovation [53]. According to previous research, an organization’s success in green innovation will be greatly enhanced if it has a higher level of GHC [53,54,55]. Research has shown that GIC is positively related to business performance in Chinese manufacturing firms. Companies with a higher level of green human capital tend to have higher levels of environmental performance, which in turn leads to improved business performance [52,56,57]. Therefore, it is asserted that, by investing in the knowledge, skills and abilities of its employees, a business can reduce its environmental impact, lower its costs and improve its reputation, all of which can contribute to its green innovation and long-term sustainability. Hence, it is proposed that:

**H_2a_.**  *GHC has a positive influence on business sustainability.*

**H_2b_.**  *GHC has a positive influence on green innovation.*

#### 2.3.2. Green Relational Capital (GRC)

GRC refers to the interactive relationships between a company and its key stakeholders in managing the corporate environment and green innovation in order to generate value and gain a competitive advantage [57]. Chen [47] used the term “green relational capital” to describe the intangible assets that a company can acquire through its relationships with customers, suppliers and partners to improve business sustainability. GRC creates strong relationships with suppliers, regulatory agencies and other stakeholders; a company can better understand the needs and preferences of these groups, which can help them to develop new products or services that meet these needs [58,59,60]. This can also lead to the development of new partnerships and collaborations, which can provide new opportunities for growth and expansion [45]. GRC can promote the sharing of innovative knowledge and contribute to the success of environmentally friendly innovations [44]. Thus, businesses with GRC can collaborate to develop new environmental technologies, ideas and opportunities [24]. Furthermore, GRC makes it easier to communicate and disseminate environmental policies throughout the organization in order to foster green innovation [56,60]. Recently, researchers have confirmed the positive relationship between GRC and firm GIN [24,61]. Hence, it is proposed that:

**H_3a_.**  *GRC has a positive influence of business sustainability.*

**H_3b_.**  *GRC has a positive influence on green innovation.*

#### 2.3.3. Green Structural Capital (GSC)

The term “green structural capital” (GSC) refers to a company’s trademarks, intellectual properties, management philosophy, organizational culture and ability to incorporate green innovation into its business operations [45]. It also refers to the company’s image and organizational culture toward environmental protection [55]. Structural capital improves a company’s processes and systems, allowing it to gain technical expertise and develop organizational capabilities [26]. Green structural capital can help businesses meet regulatory requirements, improve their reputation and public image and attract environmentally conscious customers and investors [43]. However, the impact of green structural capital on business sustainability can also be negative if it is not properly managed. If a business over-invests in green resources that do not provide a sufficient return on investment, it can negatively impact their financial performance [59]. Researchers have posited that companies with strong green structural capital are more likely to adopt and invest in new technologies and practices that reduce their environmental footprint [45]. This can include activities such as adopting renewable energy sources, implementing waste reduction strategies and developing eco-friendly products and services [55]. GSC benefits organizations by assisting them in becoming more environmentally conscious, sustainable businesses, providing the business with a strategy for environmentally friendly manufacturing and product development [59]. It is asserted that GSC in manufacturing organizations positively impacts green innovation. Hence, it is proposed that:

**H_4a_.**  *GSC has a positive influence on business sustainability.*

**H_4b_.**  *GSC has a positive influence on green innovation.*

### 2.4. Mediating Effect of Green Innovation (GINV)

The adoption of GIN may provide businesses with a competitive advantage over their rivals [24]. GIN makes it easier for organizations to implement timely policies that support their environmental strategy [18,61]. Researchers have noted that GIN strategies in business increase material efficiency and reduce disposal and waste costs [22]. Successful GIN strategy can improve business performance by reducing costs, increasing competitiveness, improving reputation, retaining talent and complying with regulations [22,24]. Hence, it is proposed that:

**H_3_.**  *Green innovation has a positive influence on business sustainability.*

GHC is important for businesses because it includes knowledge, skills and abilities to innovate and develop new green technologies and products and implement best practices for reducing environmental impact [60]. By investing in GHC, businesses can improve their overall sustainability and reduce their environmental footprint, which can lead to cost savings and increased competitiveness [40]. GHC helps businesses identify and implement green innovation to meet new environmental demands and sustain green performance [62]. Hence, it is proposed that:

**H_4a_.**  *Green innovation will mediate the relationship between GHC and business sustainability.*

GRC encourages collaboration with external partners to stimulate new ideas and creativity toward green innovation [62]. GRC promotes GIN by lowering the cost of search, information and transaction [61]. Organizations that use GRC strategies in their environmental practices are better able to create green innovations to reduce environmental impacts and attract customers that are looking for businesses with better environmental performance [52]. Organizations need to integrate the environmental knowledge produced and shared by GRC to increase the importance and use of green knowledge in order to promote green innovation [23]. Then, through green innovation, GRC can produce more sustainable business performance [23,24]. Hence, it is proposed that:

**H_4b_.**  *Green innovation will mediate the relationship between GRC and business sustainability.*

GSC is crucial in implementing green innovation [63]. Organizations can enhance their business performance by utilizing their managerial capabilities, organizational culture and integration of environmental issues [62]. GSC constitutes unique environmental knowledge that promotes the production of innovative green products [64]. Green innovation often requires raw material improvements that positively impact the environment. Therefore, businesses implement the GSC strategy to incorporate green innovation that reduces adverse environmental impacts [65] and has a positive impact on economic performance [30]. Hence, it is proposed that:

**H_4c_.**  *Green innovation will mediate the relationship between GSC and business sustainability.*

## 3. Materials and Methods

### 3.1. Sampling

The automobile manufacturing industry in China has been chosen for the data collection because it is one of the largest contributors to environmental pollution. In addition, automobile companies are adopting innovative and diverse technologies to compete in the market [66]. In light of the growing concern for environmental damage, Chinese auto manufacturers can make significant contributions to environmental management. Past studies have also discussed the nexus between automobile manufacturing and environmental pollution [66,67]. The currently employed purposive sampling technique to select the automobile manufacturing firms and online questionnaires were sent to the HR managers to disseminate to the senior managers working in the relevant departments (Top Management, Research & Development, Marketing and other relevant departments). Managers with at least 3 years of relevant experience in automobile manufacturing firms were contacted to improve the data quality because they have higher knowledge regarding business operations. The sample size was calculated following Hair et al. [68], who recommended 5 to 10 responses per item. There were 24 finalized items, making the required sample size 240. Before data collection, a questionnaire pre-test was implemented on 6 automobile industry managers to assess the validity of the questionnaire. After ensuring the content and face validity, the questionnaire was formally distributed to the managers of automobile firms. The data collection took around eight months starting from 7 March 2022 to 21 October 2022. Researchers contacted 61 firms from different cities, but only 27 responded, with a response rate of 44.26%. The five academic experts evaluated the language, constructs’ item, questionnaire layout and suggested changes. The details of the study’s participants are shown in Table 2.

### 3.2. Research Instrument

The current study uses a closed-ended questionnaire to collect participants’ data. The questionnaire consisted of three parts. The first part of the questionnaire described the purpose of the study and the participants informed consent to answer the questions. The second and third parts of the questionnaire were related to demographic information and construct items, which were marked as compulsory to avoid missing values. The constructs items were measured using a five-point Likert scale ranging from 1 = strongly disagree to 5 = strongly agree. The scales of the instrument were adapted from past studies. The scale items and their sources are provided in Table 3. The items of the scales measurement are given in Appendix A.

## 4. Results and Analysis

This study analysed the data through Partial Least Squares (PLS)-based Structural Equation Modelling (SEM). Due to the small sample size and the possibility of non-normal data, PLS-SEM was employed to assess the measurement and structural models. Hair et al. [68] suggested that PLS-SEM is a nonparametric test and does not require data normality assumption.

### 4.1. Data Examination

This research applied several data examination techniques to determine the suitability of the data. First, the Mahalanobis distance technique was applied to identify the outliers. Through this method, we identified three outliers and removed them from final analysis. Secondly, the study applies Harman’s single-factor test to test common method variance. It is essential to assess common method bias (CMB) because the data was collected through a self-reported scale; therefore, it could lead to inflation in the relationships of the study constructs, although Miller and Cardinal [69] recommended that participants’ anonymity will reflect the actual results under study. The results of the CMB indicated that a single factor had only explained 24% variance in the data, confirming that no bias exists in the data.

### 4.2. Assessment of Convergent Validity

Evaluating internal consistency is the first criterion for measuring the quality of data. This study used Cronbach’s alpha and composite reliability values to measure the data’s internal consistency. All of the constructs’ Cronbach’s alpha values exceeded the threshold value of 0.70. The more accurate criterion to evaluate the data’s internal consistency is composite reliability (CR) [70]. Therefore, CR values were also assessed to confirm the data’s internal consistency, confirming that all construct values were greater than 0.70. We then evaluated convergent validity, which measures how much one construct relates to another. This study establishes convergent validity when constructs’ average variance extracted (AVE) are greater than 0.50 and outer loadings are greater than or equal to 0.70 [71]. Table 4 shows the details of the reliability analysis and convergent validity. Table 3 shows the results of the convergent validity.

### 4.3. Discriminant Validity

The degree to which one construct is unrelated to another is referred to as discriminant validity [72]. Three criteria were used in this study to evaluate the discriminant validity. According to Fornell and Larcker [73], criterion, discriminant validity establishes when the square of AVEs values must be greater than the corresponding correlations. As shown in Table 5, the Fornell and Larcker [73] criterion was used in this study to confirm discriminant validity. The second method is Hetero-trait–Mono-trait Ratio (HTMT). The construct values for HTMT must be less than 0.85 to establish discriminant validity [74]. Table 6 illustrates that all construct values are less than 0.85. The third method is that of cross-loading values. This method also confirms discriminant validity as each of its construct’s items has greater cross-loading values than other constructs [70], as shown in Table 7.

### 4.4. Predictive Power of the Inner Model

The value of cross-validated redundancy, which determines the predictive relevance of the model (Q2), and the coefficient of determination (R2) are the two methods used to evaluate the criterion for inner model fit [70,75]. R2 value is the variation in endogenous constructs that is explained by exogenous constructs. The value of R2 for the endogenous constructs green innovation and business sustainability are 37.1% and 10.3%, respectively. The blindfolding method assessed the predictive relevance of the model (Q2). Model predictive relevance is indicated by a value of (Q2) greater than zero. The results show that (Q2) for the endogenous constructs have a value of 28.1% and 8.6% for the endogenous constructs green innovation and business sustainability, respectively, which are significantly predictive of relevance for the study’s suggested model.

### 4.5. Out of the Sample, Predict Power

In order to evaluate the PLS model’s prediction, we ran PLSpredict with 10 folds and 10 repetitions [76]. Due to asymmetric error distribution, we based our predictive evaluation on mean absolute error (MAE). Then, we compared the PLS-MAE endogenous constructs’ items with the linear model MAE (LM-MAE) endogenous constructs’ items. Most PLS-SEM indicators (endogenous items) have lower values than LM-MAE, representing medium to high predictive power. In addition, the higher values of PLS-Q2_predict for the endogenous items are desirable for PLS-SEM over LM. The results indicate that PLS-Q2_predict has higher values than LM-Q2_predict values, confirming the dominance of the PLS-SEM model. Table 8 shows the results of the assessment of PLSpredict power.

### 4.6. Hypotheses Testing

The PLS-SEM has been used with the 2000 bootstrapping sampling method to test the proposed hypotheses [77]. The proposed hypotheses’ findings showed that H1a positive impact of ECSR on GHC was insignificant (β = 0.103; t = 1.379; *p* > 0.05). H1b and H1c proposed positive impact ECSR on GRC and GSC was supported (β = 0.229; t = 3.387; *p* < 0.05) and (β = 0.173; t = 2.138; *p* < 0.05), respectively. H2a, H2b, H2c proposed positive impact of GHC, GRC and GSC on GIN were supported (β = 0.467; *t* = 8.469; *p* < 0.05), (β = 0.269; t = 4.052; *p* < 0.05) and (β = 0.145; t = 2.225; *p* < 0.05), respectively. H3 proposed a positive impact of GIN on BUS was supported (β = 0.321; t = 4.760; *p* > 0.05). The results of the direct path analysis show that GHC and GRC have the strongest impact on GIN. Similarly, GIN has a strong effect on BUS. Table 9 shows the hypotheses assessment results.

### 4.7. Mediation Analysis

The mediation analysis was carried out using the methodology proposed by Preacher and Hayes [78], in which 2000 bootstrapping was performed to determine the indirect relationships. The criteria for accepting and rejecting hypotheses were based on t-values (>1.96) and the absence of “0” in the confidence interval. According to the study’s findings, GIN partially mediates between GHC and BUS as the direct effect between GHC and BUS was significant. However, GIN fully mediates between GRC and BUS and GSC and BUS relationships. Table 10 shows the mediation results.

## 5. Discussion and Conclusions

The current study aims to analyze the impact of ECSR and GIC on organizations’ green innovation and business sustainability in the automobile manufacturing industry in China. In order to combat global warming, Chinese businesses have begun putting more emphasis on corporate environmental responsibility practices [79] and developing GIC to foster green innovation. Past studies indicate that manufacturing industries are one of China’s major sources of carbon emissions [79,80]. Therefore, this study proposes a strategy that focuses on ECSR to increase the investment in green intellectual capital and the consequent improvement of green innovation and business sustainability. The findings of the study results are summarized in Table 8 and Table 9.

The study findings revealed that ECSR significantly influences two components of GIC (GRC and GSC) in manufacturing industry in China. These findings corroborate with the previous study that ECSR significantly impacts GRC and GSC [37]. This shows that automobile manufacturers in China will invest more in GRC and GSC when its ECSR practices are higher. Concerning this, researchers argued that businesses that focus more on environment protection would allocate more resources to green management activities [79]. However, the impact of ECSR on GHC was insignificant and contradicted the findings of the previous studies [43]. The study’s findings further confirm the positive influence of the components of green intellectual capital (GHC, GRC and GSC) on green innovation. The findings of this study are consistent with previous studies that confirmed the positive influence of GIC components of GIN [35,59]. These results show that green intellectual capital has a significant role in the promotion of green innovation strategies. Therefore, companies should place more emphasis on investments in green intellectual capital to meet market demands. The positive and significant impact of green innovation on business sustainability are consistent with previous studies [24,62]. The findings of the study also confirm the positive and significant influence of GHC on business sustainability and matches the findings of the previous researchers [24]. However, the direct impacts of GRC and GSC on business sustainability were insignificant. One of the main reasons for the insignificant impact of relational and green structural capital on business sustainability may be that automobile manufacturers in China may not be able to effectively communicate the value of their green initiatives to stakeholders. If stakeholders are not aware of the business’s efforts to be more environmentally friendly, they may not be willing to pay a premium for its products.

Further, our results on the mediating role of green innovation are significant, since this mediation has been largely neglected by previous researchers, who focused on the direct impact of GIC on BUS. The findings reveal the partial mediating effect of GIN between GHC and BUS. This shows that GHC might have direct or indirect impact on BUS. The direct impact of GHC on BUS has been explored by previous researchers [24], but our findings also support the partial mediating effect of GIN in the automobile manufacturing industry. In addition, our study findings revealed that GIN fully mediates between GRC and BUS and GSC and BUS relationships. GIN as a full mediator signifies the key link to leverage green relational and green structural capital in order to achieve better business outcomes. It shows that, by adopting green practices, manufacturing organizations not only benefit the environment, but also increase their efficiency and profitability.

The current study has comprehensively focused on green management practices in the automobile manufacturing industry in China. This study incorporated the firm’s ECSR, GIC, GIN and business sustainability. The relationship between ECSR and GIC from the perspective of manufacturing firms has rarely been studied. This study presents a new aspect in evaluating manufacturing firms’ innovation and business sustainability. The automobile industry has been selected because this industry is one of the industries highly contributing to environmental pollution. To combat this and promote business sustainability, businesses’ green intellectual capital (GIC) and green innovation play a significant role. Therefore, the study analyzed the causal relationship between ECSR, GIC, GIN and BUS. The data for automobile manufacturing managers was collected and analyzed via PLS-SEM. This study also measured the predictive power of the PLS-SEM using PLSpredict. The findings of the PLSpredict revealed that PLS has better predictive power than LM. The results of the structural model supported the proposed hypotheses. However, the study found that ECSR has an insignificant impact on GHC. The study findings also confirm the mediating impact of green innovation, which has been ignored by previous researchers [24]. The significant impact of green innovation on business sustainability implies that green innovation is one of the important strategic decisions for the operations of manufacturing firms. Finally, to realize the goals of ECSR, firms need to invest more in green intellectual capital (GIC) and foster green innovation, which ultimately leads to business sustainability and competitive edge.

## 6. Implications

This study theoretically contributes to the environmental management literature and offers useful practical implications. The theoretical framework for this study broadens our knowledge of environmental management through a novel theoretical relationship between ECSR and GIC and its impact on outcomes (green innovation and business sustainability). The study findings demonstrate the importance of GIC for green innovation and business sustainability. There are several specific contributions of the current study. First, it emphasizes the importance of ECSR in management practices [59]; the current study clearly explains the role of ECSR in improving GRC and GSC. This signifies the importance of ECSR as an integrating mechanism when implementing firms’ environmental strategy. Second, the study reveals the significance of GIC in green innovation and business sustainability, extending the GIC body of knowledge. These results reinforce that GIC components (GHC, GRC, GSC) are vital in adopting green practices and prompting business sustainability. Previous researchers have emphasized the importance of mediating the effect of green innovation on business performance, but it has received little attention in previous studies [81]. The current research verified the significance of mediating the impact of green innovation.

Practically, this study has numerous implications for the managers of manufacturing firms. China’s automobile manufacturing industry plays a vital role in the development process and growth that cannot be ignored [82]. Despite significant environmental pollution and resource depletion increases, China’s economy has grown rapidly in the past several years [83]. Many Chinese companies have realized the critical need to adopt a green innovation strategy to deal with urgent environmental challenges and the country’s strict environmental regulations [47]. Businesses have started implementing environmental protection policies as ECSR has become crucial. This study depicts that firms’ ECSR policies are essential for developing GRC and GSC. However, the impact of ECSR on GHC was insignificant. To develop GHC, firms need to incorporate environmental thinking in their policies because environmental knowledge is crucial for the firms to promote green innovation.

Additionally, firms must ensure that every element of their operations complies with environmental laws and that the idea of sustainable development is ingrained in their corporate culture. The significant impact of GIC on GIN suggests that green innovation strategies are likely to be a valuable strategic choice for the operations of manufacturing firms. Therefore, it is suggested that managers should communicate with the key stakeholders to facilitate the exchange of environmental knowledge and promote firms’ green innovation. The positive influence of the components of GIC (GHC, GRC and GSC) on business sustainability signifies that green intellectual capital has a vital role in manufacturing firms’ development and growth. Therefore, policy-makers must develop green intellectual capital to accelerate the organization’s growth and contribute to environmental sustainability. It is also necessary to position environmental concern as an integrated part of firms’ strategy. In this way, businesses can encourage green innovation to achieve better business performance by prioritizing the environment.

Based on the study findings, the following recommendations are put forward for promoting environmental sustainability in China. Specifically, it includes these major aspects: first, from the perspective of green innovation, to encourage firms that have a high impact on environmental sustainability to comply with sustainable practices in the search for higher economic growth in China. Previous researchers posited that adopting green innovation helps reduce carbon emissions through less energy consumption and promotes waste recycling that ultimately improves environmental health [84,85,86]. Secondly, the Chinese government should encourage the development of a culture within organizations that reduces environmental effects. Manufacturing firms must concentrate on environmental regulations and put in place a green innovation strategy that might aid an organization’s sustainable growth. To achieve the goals of economic benefits and environmental sustainability goals, measures to reduce the consumption of natural resources such as soil, water and energy may be taken at the organization’s executive level. Finally, the current study’s findings provide a novel approach to driving environmental conservation via policy implementations. Managers should focus on creative concepts for green processes since our findings indicate that ECSR affects GRC and GSC and increases green innovation. Therefore, manufacturing firms may develop and practice innovative ideas to improve their green innovation. As a result of green innovation, firms may enhance their ability to compete and maintain business sustainability.

## 7. Limitations and Future Research

Despite the significant contributions made by this study, there are several limitations that should be considered in further research. The fact that our sample was only comprised of employees working in China’s automotive industry may restrict the results’ generalizability to other regions. To overcome this limitation, future studies may include larger sample sizes from different countries. Third, we did not take R&D spending into account when examining the impact of green innovation. Since R&D spending has been seen as essential to green innovation Wang and Juo 2021 [22], future studies may control for the R&D spending to better predict the degree to which innovation contributes to an organization’s business sustainability. Fourth, past researchers have argued that the organization’s performance depends upon its size [87]. Future studies may focus on whether small organizations perform better with less investment in GIC and green innovation. Lastly, other factors such as environmental uncertainty, learning directed toward innovation and market size may also affect organizations’ innovation and performance and should be considered in future studies.

## Figures and Tables

**Table 1:** Key findings of related studies.

Author(s)	Year	Methodology	Organization Type	Key Findings
Padilla-Lozano and Collazzo [40]	2022	Quantitative	Manufacturing industry	CSR and green innovation significantly influence competitiveness.
Marco-Lajara, et al. [18]	2022	Quantitative	Wine industry	GIC has a positive significant impact on GIN performances. CSR and knowledge management mediates the relationship between GIC and GIN.
Mehmood and Hanaysha [19]	2022	Qualitative	Systematic literature review	Study developed a research model involving the antecedent and consequences of the GIC. CSR was identified as main antecedent of GIC and GIN. GIN was also identified the consequence of GIC.
Shiekh [41]	2022	Quantitative	Manufacturing companies	GHC and GSC have positive significant impact on social innovation. GRC has insignificant impact on social innovation.
Ali et al. [23]	2021	Quantitative	Manufacturing SMEs	GHC and GSC increases GIN. GRC has insignificant impact on GIN.
Liao et al. [36]	2021	Quantitative	High-tech industries	GHC and GRC were positively related to the perception of CSR. Moreover, the perception of CSR mediated the associations between GHC, GRC and employees’ pro-environmental behavior.
Shah et al. [42]	2021	Quantitative	Service industry (hotels)	GHC, GRC and GSC have positive significant impact on environmental responsibility.
Ullah et al. [24]	2021	Quantitative	Manufacturing companies	GHC, GRC and GSC have positive significant impact on business sustainability. Information technology (IT) capability moderates the relationship between GIC and business sustainability.
Wang and Juo [22]	2021	Quantitative	High-tech firms	GIN fully mediates the relationships GHC–conomic performance and GSC–green performance and partially mediates the relationship between GRC–economic performance and GRC–green performance.
Kraus et al. [34]	2020	Quantitative	Manufacturing companies	CSR has no direct significant influence on environmental performance, but it has positive significant on GIN and environmental strategy.
Li et al. [25]	2020	Quantitative	Manufacturing companies	GIN has significant impact on business Sustainability.
Sudibyi and Sutanto [43]	2020	Quantitative	Manufacturing companies	CSR has a positive impact on three components of GIC (GHC, GRC, GSC). Environmental consciousness has positive impact on GHC and GSC but not on GRC.
Yong et al. [21]	2020	Quantitative	Manufacturing companies	Analyzed the impact of green HRM on business sustainability. Green recruitment and green training have positive effects on sustainability.
Omar et al. [44]	2019	Quantitative	Manufacturing SMEs	GHC has insignificant impact on organizational learning while GRC and GSC have positive significant impact on organizational learning. Organizational learning has a positive significant impact on business sustainability.
Yusoff et al. [45]	2019	Quantitative	Manufacturing SMEs	The results show that GSC and GRC have positive relationship with business sustainability, while GHC did not.
Chuang and Huang [35]	2018	Quantitative	Manufacturing companies	ECSR has significant positive impact on GIC components. GSC and GRC have positive impact on EP and business competitiveness (BC). EP has positive impact on BC.
Delgado-Verde et al. [46]	2014	Quantitative	Manufacturing industry	Green organizational capital has no direct effect on environmental product innovation. Social capital has positive significant impact on environmental product innovation. Green social capital mediates the relationship between green organizational capital and environmental product innovation.
Chen and Chang [20]	2013	Quantitative	Manufacturing industry	Corporate environmental ethics has positive impact on green relationship learning and green innovation performance. GHC mediates between corporate environmental ethics and green relationship performance.

Note: CSR = corporate social responsibility; GIC = green intellectual capital; GHC = green human capital; GRC = green relational capital; GSC = green structural capital; GIN = green innovation; EP = environmental performance.

**Table 2:** Participants’ profile.

		Frequency	Percentage
Gender	Male	141	50.5%
	Female	96	40.5%
Age	Under 30 years	51	21.5%
	30 to 40 years	68	28.7%
	More than 40 years	118	49.8%
Qualification	Diploma	31	13.1%
	Graduate	59	24.9%
	Postgraduate	107	45.1%
	Others	40	16.9%
Title	Chief executive officer	4	1.7%
	Managing director	17	7.2%
	R & D Manager	55	23.2%
	Marketing manager	74	31.2%
	Others	87	36.7%
Experience	3 to 7 years	58	24.5%
	8 to 12 years	44	18.6%
	13 to 17 years	74	31.2%
	18 to 22 years	59	24.9%
	More than 22 years	2	0.8%

**Table 3:** Constructs’ measurement sources.

Constructs	Items	Sources
Environmental Corporate Social Responsibility	5	Bacinello et al. [33]
Green Human Capital	3	Yong et al. [21]; Chen [47]
Green Relational Capital	3	Ullah et al. [57]; Chen [47]
Green Structural Capital	5	Wang and Juo [22]
Green Innovation	4	Wang and Juo [22]
Business Sustainability	4	Ullah et al. [57]

**Table 4:** Descriptive Analysis and Measurement Model.

Constructs	Items	Loading	α	CR	AVE
Environmental corporate social responsibility	ECSR1	0.835	0.913	0.935	0.742
	ECSR2	0.855			
	ECSR3	0.901			
	ECSR4	0.858			
	ECSR5	0.856			
Green human capital	GHC1	0.783	0.760	0.860	0.673
	GHC2	0.849			
	GHC3	0.827			
Green relational capital	GRC1	0.881	0.829	0.898	0.747
	GRC2	0.801			
	GRC3	0.907			
Green structural capital	GSC1	0.947	0.963	0.971	0.871
	GSC2	0.945			
	GSC3	0.961			
	GSC4	0.887			
	GSC5	0.926			
Green innovation	GINV1	0.849	0.901	0.931	0.772
	GINV2	0.914			
	GINV3	0.848			
	GINV4	0.930			
Business sustainability	BUS1	0.942	0.947	0.962	0.862
	BUS2	0.917			
	BUS3	0.919			
	BUS4	0.936			

Note: ECSR = Environmental corporate social responsibility, GHC = Green human capital, GRC = Green relational capital, GSC = Green structural capital, GINV = Green innovation, BUS = Business sustainability.

**Table 5:** Discriminant Validity (Fornell and Larcker criterion).

Latent Variables	1	2	3	4	5	6
Environmental corporate responsibility	**0.861**					
Green human capital	0.103	**0.820**				
Green relational capital	0.229	0.125	**0.864**			
Green structural capital	0.173	0.063	0.257	**0.933**		
Green innovation	0.094	0.509	0.365	0.243	**0.879**	
Business sustainability	0.142	0.490	0.056	0.066	0.321	**0.929**

Note: The diagonals (in bolds) represent the square root of AVE and off-diagonal values represent the correlations of each construct with other constructs. All correlations are statistically significant (*p* < 0.01).

**Table 6:** Discriminant Validity Hetero-trait–Mono-trait Ratio (HTMT).

Latent Variables	1	2	3	4	5	6
Business sustainability						
Environmental corporate responsibility	0.152					
Green human capital	0.566	0.120				
Green innovation	0.346	0.114	0.606			
Green relational capital	0.064	0.261	0.156	0.419		
Green structural capital	0.069	0.177	0.083	0.260	0.289	

**Table 7:** Cross-loading.

	BUS	ECSR	GHC	GINV	GRC	GSC
BUS1	**0.942**	0.122	0.449	0.288	0.044	0.063
BUS2	**0.917**	0.140	0.468	0.325	0.033	0.041
BUS3	**0.919**	0.141	0.464	0.277	0.065	0.092
BUS4	**0.936**	0.125	0.440	0.299	0.067	0.054
ECSR1	0.109	**0.835**	0.047	0.041	0.210	0.073
ECSR2	0.114	**0.855**	0.092	0.026	0.179	0.099
ECSR3	0.091	**0.901**	0.082	0.099	0.246	0.144
ECSR4	0.161	**0.858**	0.105	0.137	0.196	0.175
ECSR5	0.135	**0.856**	0.110	0.082	0.152	0.228
GHC1	0.338	0.063	0.783	0.335	0.114	0.074
GHC2	0.533	0.077	0.849	0.477	0.142	0.055
GHC3	0.313	0.111	0.827	0.423	0.051	0.033
GINV1	0.316	0.090	0.430	**0.903**	0.375	0.171
GINV2	0.210	−0.014	0.424	**0.848**	0.368	0.249
GINV3	0.312	0.121	0.475	**0.914**	0.329	0.234
GINV4	0.288	0.131	0.463	**0.849**	0.204	0.203
GRC1	0.050	0.188	0.124	0.330	**0.881**	0.249
GRC2	0.044	0.232	0.107	0.303	**0.801**	0.134
GRC3	0.050	0.169	0.091	0.309	**0.907**	0.288
GSC1	0.085	0.189	0.068	0.234	0.268	**0.947**
GSC2	0.039	0.147	0.036	0.223	0.249	**0.945**
GSC3	0.089	0.188	0.078	0.234	0.238	**0.961**
GSC4	0.051	0.153	0.027	0.179	0.205	**0.887**
GSC5	0.039	0.128	0.079	0.258	0.237	**0.926**

**Table 8:** Assessment of PLSpredict power.

Items	PLS-MAE	LM-MAE	PLS (MAE)-LM (MAE)	PLS-Q^2^_Predict	LM-Q^2^_Predict	PLS-Q^2^_Predict- LM-Q^2^_Predict
BUS1	0.755	0.760	**−0.005**	0.007	−0.020	**0.027**
BUS4	0.750	0.755	**−0.005**	0.007	−0.016	**0.023**
BUS3	0.723	0.728	**−0.005**	0.009	−0.013	**0.022**
BUS2	0.701	0.701	**0.00**	0.008	−0.010	**0.018**
GHC3	0.724	0.725	**−0.001**	0.001	−0.024	**0.025**
GHC2	0.512	0.520	**−0.007**	−0.004	−0.031	**0.027**
GHC1	0.711	0.703	0.007	−0.004	−0.007	**0.003**
GINV2	0.559	0.567	**−0.009**	−0.019	−0.023	**0.004**
GINV1	0.512	0.527	**−0.015**	0.002	−0.019	**0.021**
GINV3	0.497	0.516	**−0.019**	0.008	−0.011	**0.019**
GINV4	0.577	0.602	**−0.025**	0.012	−0.007	**0.019**
GRC2	0.624	0.634	**−0.011**	0.043	0.011	**0.032**
GRC3	0.619	0.634	**−0.015**	0.015	−0.016	**0.031**
GRC1	0.547	0.561	**−0.014**	0.023	−0.009	**0.032**
GSC1	0.513	0.507	0.006	0.020	0.012	**0.008**
GSC2	0.515	0.507	0.007	0.006	−0.002	**0.008**
GSC5	0.500	0.496	0.004	0.001	−0.006	**0.007**
GSC3	0.510	0.504	0.006	0.019	0.009	**0.01**
GSC4	0.524	0.529	**−0.005**	0.009	−0.027	**0.036**

Note: The bold values are the difference between PLS and LM model indicating that PLSpredict model has better prediction power than LM.

**Table 9:** Hypotheses Assessment Summary.

Hypotheses	Beta	*p*-Values	t-Values	Decision
H1a: ECSR → GHC	0.103	0.168	1.379	Not supported
H1b: ECSR → GRC	0.229	0.001	3.387	Supported
H1c: ECSR → GSC	0.173	0.033	2.138	Supported
H2a: GHC → BUS	0.451	0.000	6.012	Supported
H2b: GHC → GIN	0.467	0.000	8.469	Supported
H3a: GRC → BUS	−0.045	0.520	0.643	Not supported
H3b: GRC → GIN	0.467	0.000	8.469	Supported
H4a: GSC → BUS	0.025	0.668	0.429	Not supported
H4b: GSC → GIN	0.145	0.026	2.225	Supported
H5: GIN → BUS	0.321	0.000	4.760	Supported

**Table 10:** Mediation results.

Hypotheses	Beta	*p*-Values	t-Values	C.I	Decision
H6a: GHC → GIN → BUS	0.150	0.000	3.529	0.072, 0.237	Partial Mediation
H6b: GRC → GIN → BUS	0.086	0.001	3.212	0.039, 0.145	Full Mediation
H6c: GSC → GIN → BUS	0.046	0.029	2.192	0.010, 0.095	Full Mediation

Note: Path coefficients (Beta); significant at *p* < 0.05.

## Data Availability

The datasets analyzed during the current study are available from the corresponding author on reasonable request.

## Appendix A

- **Environmental Corporate social responsibility**

ECSR1: Our firm compliance with environmental legislation.

ECSR2: Our firm uses clean technologies.

ECSR3: Our firm ensures sustainable use of natural resources.

ECSR4: Our firm has a waste management policy.

ECSR5: Our firm conforms requirements of environmental management.

- **Green human Capital**

GHC1: The sustainability of our firm environment is dependent on the competencies and skills of the organization’s employees.

GHC2: To enhance the long-term sustainability, our firm include green human capital into their working environments.

GHC3: Green human capital contributes to the sustainability of the firm.

- **Green Relational Capital**

GRC1: Our firm designs products and/or services in compliance with the environmentalism desires of our customers.

GRC2: Customer satisfaction concerning the environmental protection of our firm is better than that of our major competitors.

GRC3: The cooperative relationships concerning the environmental protection of our firm with our upstream suppliers are stable.

- **Green structural Capital**

GSC1: The management system for environmental protection in our firm is superior to that of our major competitors.

GSC2: Investments in environmental protection facilities in our firm are more than those of our major competitors.

GSC3: Competence in developing green products in our firm is better than that of our major competitors.

GSC4: The overall operational processes for environmental protection in our firm work smoothly.

GSC5: The knowledge management system for environmental management in our firm is favorable for the accumulation of the knowledge of environmental management.

- **Green Innovation**

GINV1: Our firm uses less or nonpolluting/toxic materials.

GINV2: Our firm uses eco-labeling.

GINV3: Our firm uses recycle, reuse and remanufacture material.

GINV4: Our firm uses cleaner technology to make savings and prevent pollution (such as energy, water and waste).

- **Business Sustainability**

BUS1: Business sustainability is necessary for our firm to ensure long-term growth.

BUS2: Business sustainability helps our firm to compete well in the industry.

BUS3: Sustainability increases the sales of our firm as consumers are more attracted to sustainable products.

BUS4: Sustainability helps our firm to develop long-term strategies.
